# Glycated ACE2 reduces anti-remodeling effects of renin-angiotensin system inhibition in human diabetic hearts

**DOI:** 10.1186/s12933-022-01573-x

**Published:** 2022-08-05

**Authors:** Raffaele Marfella, Nunzia D’Onofrio, Gelsomina Mansueto, Vincenzo Grimaldi, Maria Consiglia Trotta, Celestino Sardu, Ferdinando Carlo Sasso, Lucia Scisciola, Cristiano Amarelli, Salvatore Esposito, Michele D’Amico, Paolo Golino, Marisa De Feo, Giuseppe Signoriello, Pasquale Paolisso, Emanuele Gallinoro, Marc Vanderheyden, Ciro Maiello, Maria Luisa Balestrieri, Emanuele Barbato, Claudio Napoli, Giuseppe Paolisso

**Affiliations:** 1 Department of Advanced Medical and Surgical Sciences, Università degli Studi della Campania “Luigi Vanvitelli”, Piazza Miraglia, 2, 80138 Naples, Italy; 2 Mediterranea Cardiocentro, Naples, Italy; 3 Department of Precision Medicine, The University of Campania “Luigi Vanvitelli”, 80138 Naples, Italy; 4 Department of Experimental Medicine, University of Campania “Luigi Vanvitelli”, 80138 Naples, Italy; 5 Unit of Cardiac Surgery and Transplants, AORN Ospedali dei Colli-Monaldi Hospital, 80131 Naples, Italy; 6 Unit of Pathological Anatomy, Aversa Hospital, Caserta, Italy; 7 Cardiology Division, University “L. Vanvitelli” - Monaldi Hospital, 80131 Naples, Italy; 8 Department of Cardio-Thoracic Sciences, University of Campania “Luigi Vanvitelli”, Naples, Italy; 9 Statistical Unit-Department of Mental Health and Public Medicine, University of Campania “Luigi Vanvitelli”, Naples, Italy; 10 Cardiovascular Center Aalst, OLV-Clinic, Aalst, Belgium; 11 Department of Advanced Biomedical Sciences, University of Naples Federico II, Naples, Italy

**Keywords:** Heart transplantation, Diabetes, HbA1c, Diabetic cardiomyopathy, RAS-inhibition therapy

## Abstract

**Background:**

High glycated-hemoglobin (HbA1c) levels correlated with an elevated risk of adverse cardiovascular outcomes despite renin-angiotensin system (RAS) inhibition in type-2 diabetic (T2DM) patients with reduced ejection fraction. Using the routine biopsies of non-T2DM heart transplanted (HTX) in T2DM recipients, we evaluated whether the diabetic milieu modulates glycosylated ACE2 (GlycACE2) levels in cardiomyocytes, known to be affected by non-enzymatic glycosylation, and the relationship with glycemic control.

**Objectives:**

We investigated the possible effects of GlycACE2 on the anti-remodeling pathways of the RAS inhibitors by evaluating the levels of Angiotensin (Ang) 1–9, Ang 1–7, and Mas receptor (MasR), Nuclear-factor of activated T-cells (NFAT), and fibrosis in human hearts.

**Methods:**

We evaluated 197 first HTX recipients (107 non-T2DM, 90 T2DM). All patients were treated with angiotensin-converting enzyme inhibitor (ACE-I) or angiotensin receptor blocker (ARB) at hospital discharge. Patients underwent clinical evaluation (metabolic status, echocardiography, coronary CT-angiography, and endomyocardial biopsies). Biopsies were used to evaluate ACE2, GlycACE2, Ang 1–9, Ang 1–7, MasR, NAFT, and fibrosis.

**Results:**

GlycACE2 was higher in T2DM compared tonon-T2DM cardiomyocytes. Moreover, reduced expressions of Ang 1–9, Ang 1–7, and MasR were observed, suggesting impaired effects of RAS-inhibition in diabetic hearts. Accordingly, biopsies from T2DM recipients showed higher fibrosis than those from non-T2DM recipients. Notably, the expression of GlycACE2 in heart biopsies was strongly dependent on glycemic control, as reflected by the correlation between mean plasma HbA1c, evaluated quarterly during the 12-month follow-up, and GlycACE2 expression.

**Conclusion:**

Poor glycemic control, favoring GlycACE2, may attenuate the cardioprotective effects of RAS-inhibition. However, the achievement of tight glycemic control normalizes the anti-remodeling effects of RAS-inhibition.

*Trial registration*: https://clinicaltrials.gov/ NCT03546062.

**Supplementary Information:**

The online version contains supplementary material available at 10.1186/s12933-022-01573-x.

## Introduction

Therapy to inhibit the renin-angiotensin system (RAS) is effective and well-tolerated in diabetic and nondiabetic patients with heart failure [1–4], regardless of the clinical findings. However, dysglycemia is associated with a higher risk of adverse cardiovascular outcomes in type 2 diabetic (T2DM) patients than in nondiabetic patients with heart failure and reduced ejection fraction (HFrEF), independently of the medical therapy, including angiotensin-converting enzyme inhibitor (ACE-I) or angiotensin receptor blocker (ARB) [5, 6]. At present, because the RAS pathway plays a pivotal role in diabetic complications, including diabetic cardiomyopathy [7], blunted anti-remodeling effects of RAS-inhibitor therapy in T2DM patients cannot be ruled out. In particular, the link between glycemic control and the RAS-inhibition therapy is worthed to be better characterized. Indeed, it is not yet known whether a high rate of glycated hemoglobin (HbA1c, > 7%) impacts the anti-remodeling molecular pathway of RAS-inhibition therapy. Previous findings demonstrated that ACE-I and ABR exerted cardioprotective effects by promoting Angiotensin (Ang) 1–9 and Ang 1–7 upregulation derived from angiotensin-converting enzyme 2 (ACE2) activity [8]. In fact, Ang 1–9 and Ang 1–7 were shown to attenuate cardiac remodeling, reducing heart fibrosis and dysfunction through the inhibition of nuclear factor of activated T-cells (NFAT) via Mas receptor (MasR) activity [9, 10]. Hyperglycemia-mediated non-enzymatic glycation is well known to exacerbate long-term diabetic complications [11, 12] by altering molecular conformation and enzymatic activity and interfering with receptor functioning [13]. Interestingly, poor blood glycemic control was shown to correlate with an elevated risk of adverse cardiovascular outcomes despite the RAS inhibition by ACE-I or ARB therapy in dysglycemic patients with HFrEF [1]. Recent evidence in autopsy cases showed that in cardiomyocytes from autopsied and explanted hearts of T2DM, the higher expression levels of glycosylated ACE2 (GlycACE2) compared to non-T2DM subjects were attributable to non-enzymatic glycation of four lysine residues in the neck domain of ACE2 [14].

To date, no evidence has demonstrated the role of hyperglycemia on the expression of GlycACE2 in cardiomyocytes of human beating diabetic hearts, which could impact the binding of Ang-I and Ang-II and, consequently, the expression levels of Ang 1–9 and Ang 1–7 in humans. So far, in biopsies of transplanted hearts [15], we obtained insights into the effects of the RAS inhibition on human diabetic hearts and the relative roles of the diabetic milieu and glycemic control. Moreover, in the present study, we investigated whether changes in the GlycACE2 levels affect the anti-remodeling molecular pathways of the RAS inhibition, evaluating Ang 1–9 and Ang 1–7 expression levels in cardiomyocytes from hearts transplanted in T2DM patients.

## Methods

### Patients

**Fig. 1:**
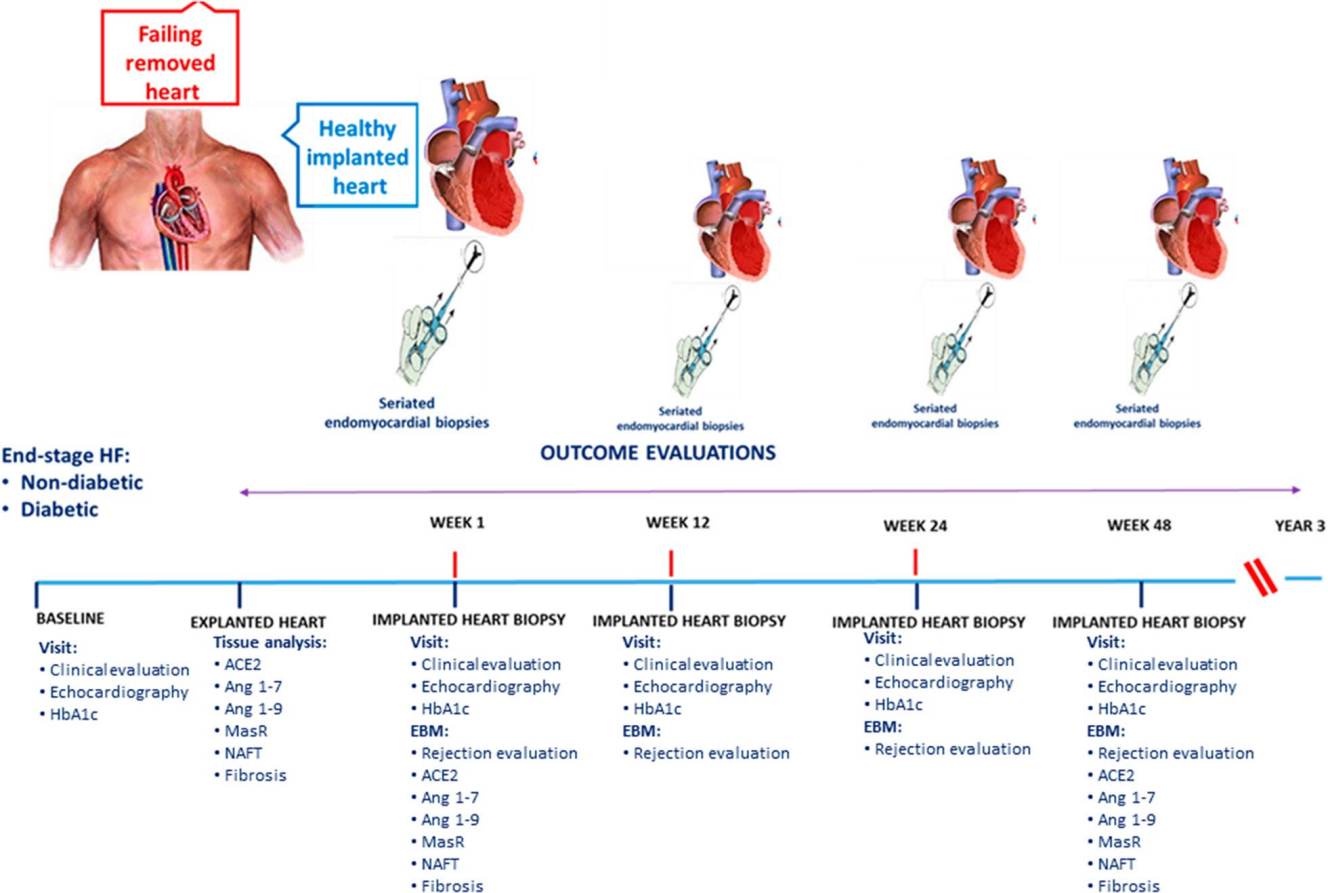
Study protocol

Since January 2010, we have been conducting a prospective study (NCT03546062) [15] under ALCOA (Attributable, Legible, Contemporaneous, Original, and Accurate) integrity protocols with a follow-up of 12 months on patients who underwent their first HTX at the HTX referral center of Monaldi Hospital (Naples, Italy) following International Society for Heart and Lung Transplantation (ISHLT) guidelines [16]. The Ethical Committee approved the study (protocol no. 438) and patients gave written informed consent. The study group consisted of 197 patients enlisted to undergo HTX and followed for 12 months (Fig. 1). All patients were treated with RAS-inhibitor drugs (ACE-I or ARB). The recipients’ patients, at baseline and follow-up, under ACE-I received either 5 mg, 10 mg or 20 mg of Lisinopril once daily and/or 5 mg, 10 mg or 20 mg of Enalapril once daily. The recipients’ patients, at baseline and at follow-up, under ARB received 50 mg, 100 mg, or 150 mg of Losartan once daily.

The study population was divided into two groups according to whether patients did or did not have T2DM before the transplantation. The study included patients with T2DM for at least 6 months before HTX, without diabetic complications, following ISHLT guidelines [16]. Patients with endomyocardial biopsy specimens consistent with ISHLT Grade 2R are considered positive for rejection, donor-specific antibodies (DSA) and IgM and IgG cytomegalovirus antibodies and increased T4/T8 ratio as well as with post-HTX diabetes were excluded from the study. Details of the surgical technique employed and the pharmacological tools at the follow-up were previously reported [15].

### Clinical and echocardiographic evaluations

The internationally accepted evaluations were recorded after HTX at weeks 1, 24, and 48 (clinical and instrumental evaluation and glycemic control, i.e., fasting glycemia and HbA1c). At 12-month follow-up, the patients were divided, as post hoc analysis, into non-T2DM, T2DM with good glycemic control (HbA1c < 7%), and T2DM in poor glycemic control (HbA1c ≥ 7%) groups, based on the mean HbA1c evaluated quarterly [17]. Moreover, Ang 1–7 and Ang 1–9 levels in urine samples by ELISA, following the manufacturer’s protocol for biological fluids (MBS703599-96 and MBS2022456, MyBioSource), were determined. 24-h urine samples were collected at weeks 1 (Basal), 12 (Intermediate), and 48 (Final) in plastic containers by adding 20 ml of 6N HCl to completely inhibit the degradation of angiotensin peptides at room temperature for over 36 h [18]. In addition, we performed echocardiographic evaluations of systolic [ejection fraction (EF) and tricuspid annular plane systolic excursion (TAPSE)] and diastolic (E/e′ ratio) heart function at baseline and after a 12-months follow-up, as previously described (Fig. 1) [19].

### Heart biopsies

After HTX, all patients’ endomyocardial biopsies (EMBs) were obtained either as a routine surveillance protocol or as tools for diagnosing allograft dysfunction and clinically suspected rejection [15, 16]. The standard biopsy schedule was performed as follows: weekly for the first month, fortnightly for the next month, once in the next 4 weeks, once in the next 6 weeks, then every 3 months for the next 2 years, and after that, every 6 months (Fig. 1). Biopsies were performed as previously described [15]. Endomyocardial biopsy specimens were analyzed for cellular viability by evaluating Hypoxia-inducible factor-1α (HIF-1α) without suspicion of histological rejection. Although the study was based on prospective biopsies of implanted hearts, an experienced thoracic surgeon excised four to six tissue specimens of about 5–10 mm^3^ from the left ventricular free wall. Tissues were immediately treated and analyzed as described previously [15].

### Tissue analysis

The biopsy evaluations were performed at 1 week (Basal) and 48 weeks (Final) (Fig. 1).

#### ACE2 expression

Immunofluorescence detection of ACE2 was evaluated in deparaffinized explanted heart sections from non-T2DM, T2DM with good glycemic control (HbA1c < 7%), and T2DM in poor glycemic control (HbA1c > 7%). Briefly, antigen retrieval buffer (10 mM Sodium citrate, 0.05% Tween 20, pH 6.0) was added to deparaffinized and rehydrated sections and boiled in the microwave for 20 min. Slides were washed in phosphate-buffered saline (PBS) followed by incubation for 30 min in Tris-buffered saline (TBS) containing 50 mM ammonium chloride to reduce background fluorescence. All sections were blocked for 1 h at room temperature (RT) in fetal bovine serum (FBS) with saponin (0.1 g/ml) and stained with primary antibodies against ACE2 (1:500, ab15348, Abcam) and Cardiac Troponin T [1C11] (1:500, ab8295, Abcam) for 16 h. Sections, incubated using Alexa Fluor 488 or 633 secondary antibodies diluted at 1:1000 in blocking solution for 1 h at RT, were then quenched for autofluorescence using the Vector TrueVIEW Autofluorescence Quenching Kit (VEC-SP-8500, Vector Laboratories). To ensure that what appears to be specific staining was not caused by non-specific interactions of immunoglobulin molecules with the sample, sections from non-T2DM and T2DM patients were incubated with blocking solution, supplemented with a non-immune immunoglobulin IgG antibody, followed by a secondary antibody incubation for 1 h at RT. All samples were stained with DAPI (4′,6-diamidino-2-phenylindole; 5 µg/ml) for 10 min before mounting in Vectashield Mounting Medium (Vector Laboratories, catalog no. H-1700). Using a Zeiss LSM 710 confocal microscope, all slides were imaged with a plan apochromat X63 (NA1.4) oil immersion objective.

#### GlycACE2 levels

The myocardial levels of GlycACE2 protein were evaluated in explanted heart samples and endomyocardial biopsies from non-T2DM and T2DM patients by immunoblotting analysis. As for the preparation of myocardial protein extracts, 2D lysis buffer (7 mol/l urea, 2 mol/l thiourea, 4% CHAPS [3-([3-cholamidopropyl] dimethylammonium)-1-propane sulfonate] buffer, 30 mmol/l Tris–HCl, pH 8.8), were added to tissues cut into small pieces. Tissues homogenized with a Precellys 24 system (Bertin Technologies) were centrifuged at 800×*g* for 10 min at 4 °C to collect the supernatant. 50–60 μg of sample proteins were separated by sodium dodecyl sulfate-polyacrylamide gel electrophoresis (SDS-PAGE) and then transferred to nitrocellulose membranes. Membranes were incubated for 1 h at RT with blocking buffer solution, TBS-T containing 20 mM Tris, pH 7.6, 100 nM NaCl, 0.1% Tween-20, and 5% non-fat dry milk under gentle shaker. Membranes were then incubated with specific primary antibodies against ACE2 (1:1000, ab15348, Abcam) or GlycACE2 (1:1000, #4355, Cell Signaling Technology) at 4 °C overnight, followed by incubation with peroxidase-conjugated secondary antibodies for 1 h at RT. In this study two antibodies have been used in order to distinguish GlycACE2 from ACE2. The antibody for ACE2 (ab15348, Abcam) detects a band size in human tissues at 120–135 kDa, as reported by the manufacturer. The antibody for GlycACE2 (#4355, Cell Signaling) detected a band at 120–135 kDa, and was also tested with an aliquot of recombinant human ACE2 (hACE2) (MW = 100 kDa) after in vitro glycation [14]. As reported, hACE2 was separated on SDS-PAGE by using 7% gels in reducing and non-reducing conditions and then transferred to nitrocellulose membrane [14]. Membrane incubated with antibody against GlycACE2 (1:1000) (#4355, Cell Signaling Technology) showed a band at a molecular weight higher than 100 kDa (about 135 kDa) supporting the non-enzymatic glycosylation of hACE2 protein. This evidence was strengthened by the detection of a band at 250 kDa under reducing conditions, corresponding to the dimer formation (Additional file 1: Fig. S1). Protein normalization was performed using α-tubulin (#2125, Cell Signaling, catalog no. 2125; 1:5000). The chemioluminescent reaction has been performed on a dried membrane to independently focus on non-glycosylated ACE2 or glycosylated ACE2 protein. Images were acquired by using Image Lab 5.2.1, Molecular Imager ChemiDoc XRS Imaging system (Bio-Rad Laboratories), and band densities were measured by ImageJ software (National Institutes of Health, Bethesda, USA) and expressed as arbitrary units (AU). The GlycACE2 content was evaluated as the percentage of the total amount of ACE2.

#### Real time-polymerase chain-reaction

Total RNA was isolated from human heart sample homogenates, according to the manufacturer’s protocol, by using RNeasy Mini kit (74106, Qiagen) and was quantified with NanoDrop 2000c Spectrophotometer (Thermo Fisher Scientific). Genomic DNA (gDNA) contaminations were removed from heart samples and mRNA was converted to cDNA by using QuantiTect Reverse Transcription kit (205311, Qiagen)—Reverse Transcription with Elimination of Genomic DNA for Quantitative, Real-Time PCR Protocol—and Gene AMP PCR System 9700 (Applied Biosystems). cDNA were amplified with the CFX96 Real-time System C1000 Touch Thermal Cycler (BIORAD), according to the protocol “Two-Step RT-PCR (Standard Protocol)”. Particularly, QuantiTect SYBR Green PCR Kit (204143, Qiagen) and QuantiTect Primer Assays were used in order to detect human ACE2 (ACE2—QT00034055, Qiagen) gene expression, quantized with 2^−ΔΔCt^ method by using GAPDH (QT00079247, Qiagen) as control.

#### Ang 1–7, Ang 1–9, MasR, and NFAT

Enzyme-linked immunosorbent assay (ELISA) colorimetric kits were used for the determination of human Ang 1–7, Ang 1–9, *MasR* and NFAT (Human Ang 1–7 ELISA Kit, E-EL-H5518, Elabscience; Human Ang 1–9 ELISA Kit, EKU10061, Biomatik; Human MAS1 ELISA Kit, abx555483, Abbexa; Human NFAT activation molecule 1 (NFAM1) ELISA Kit, abx520337, Abbexa) levels in tissue extracts from heart biopsies (1 mg/ml of total protein), according to the manufacturer’s protocol for tissue homogenates. Briefly, tissues were rinsed in ice-cold PBS, cut into small pieces, and homogenized in fresh 2D lysis buffer with a Precellys 24 system (Bertin Technologies). The resulting suspension was centrifuged for 5 min at 10,000×*g* and the clarified surnatant was incubated in the pre-coated plates with specific anti-Ang 1–7, -Ang 1–9, -MAS1 and -NFAM1 antibodies, following the manufacturer's instruction. For each sample, the Optical Density (OD) is measured spectrophotometrically at 450 nm in a microplate reader (Bio-Rad) and Ang 1–7, Ang 1–9, MAS1 and NFAM1 levels in samples determined by plotting the absorbance values against concentrations of each standard curve. The assessment of Ang 1–7 and Ang 1–9 content was performed by using ELISA kits with high specificity in the detection to avoid significant cross-reactivity or interference between Ang 1–7 or Ang 1–9 and their analogs as reported in the specific datasheet. In detail, during the reaction, human Ang 1–7 or Ang 1–9 in samples compete with a fixed amount of human Ang 1–7 or Ang 1–9 on the solid phase supporter for sites on the biotinylated detection Ab specific to Human Ang 1–7 or Ang 1–9. No significant cross-reactivity or interference between human Ang 1–7 and Ang 1–9 was observed.

#### Fibrosis evaluation

For morphological diagnosis, sections (4 μm thick) were stained with hematoxylin and eosin (H&E). Masson's Tricromica Stain was used for the differential staining of collagen. All stained samples were examined under light and digital microscopes. The content of collagen fibers relative to the total adjacent normal tissue by image analysis using the software Zen 3.3 (blue edition, Zeiss) was also evaluated.

### Statistical analysis

Data are expressed as mean ± SD for continuous variables and percentage for categorical variables.Two-way repeated-measures ANOVA was conducted to determine the differences in cardiac GlycACE2, Ang 1–7, Ang 1–9, MasR, and NFAT levels at baseline and after 12 months in diabetic and non-diabetic patients. Interaction effect was assessed to determine within-group changes and between-group differences at baseline and 12 months. The Shapiro–Wilk test was used to assess the normality of the data. A multiple regression model was used to assess changes in GlycACE2 levels by age, sex, BMI, and glycated hemoglobin levels. A P-value < 0.05 was considered statistically significant. Data were analyzed with SPSS software (version 23).

## Results

### Baseline characteristics and outcomes at 1-year follow-up.

**Fig. 2:**
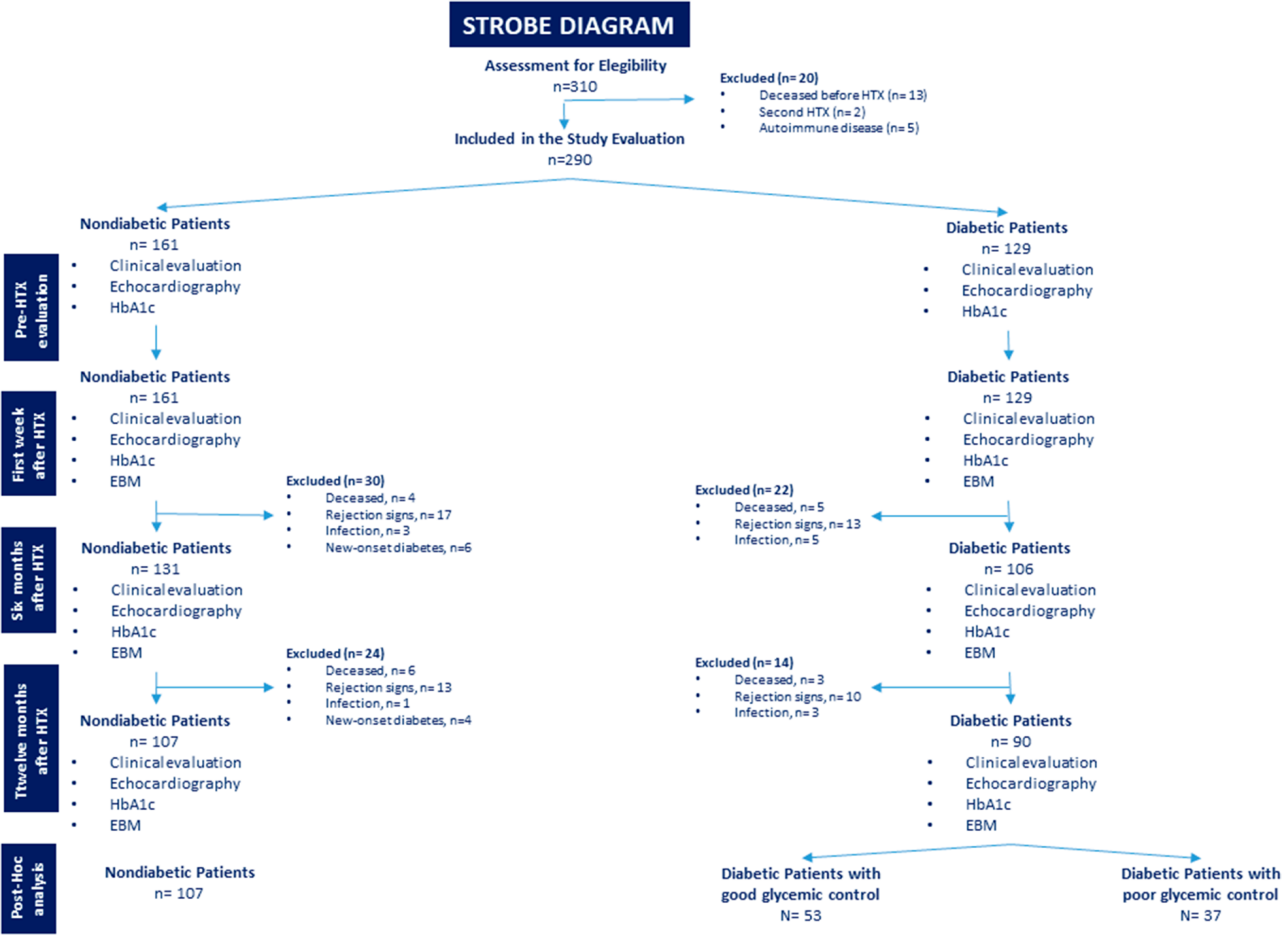
Flow-chart of the study protocol

**Fig. 3:**
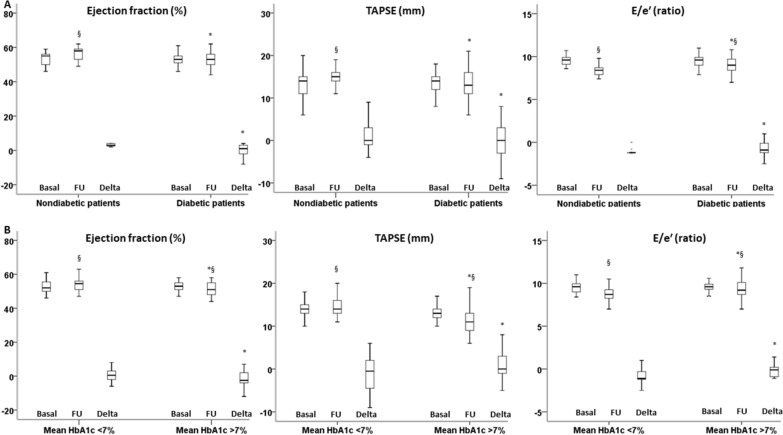
Ejection fraction, tricuspid annular plane systolic excursion (TAPSE) and the ratio of transmitral Doppler early filling velocity to tissue Doppler early diastolic mitral annular velocity (E/e′) and cardiac at week 1 (Basal) and week 48 (follow-up) from HTX with respective Delta values, in nondiabetics and diabetic patient (**A**), and in diabetic patients in good glycemic control (< 7%) and poor glycemic control (≥ 7%). (Boxplot, a plot type that displays the median, 25th, and 75th percentiles and range). *P < 0.05 vs nondiabetics, ^§^P < 0.05 vs basal values (**A**). *P < 0.05 vs diabetic patients in good glycemic control (< 7%), ^§^P < 0.05 vs basal values

Characteristics of the HTX recipients and donors are shown in Table 1. At baseline, recipients were divided into two groups: those without (n = 161, 55%) and those with (n = 129, 45%) T2DM. No significant difference was found in 1-year mortality between groups (10 non-T2DM patients, 6.3%; 8T2DM patients, 6.2%). No significant difference was seen at 1-year rejection complication (30 non-T2DM patients, 19.0%; 23 T2DM patients, 17.8%) and infection (4 non-T2DM patients and6T2DM patients). Since 10 normal recipients developed new-onset diabetes, the study population included 107 (54%) non-T2DM and 92 (46%) T2DM recipients (Fig. 2). Compared with patients without pretransplant T2DM, patients who had pretransplant diabetes spent significantly more time in the hospital during the first 1 year after HTX. The mean time hospitalized was 21 days (median, 15 days; maximum, 70 days) for recipients who had pretransplant T2DM and 18 days for recipients who did not (median, 13 days; maximum, 69 days). T2DM patients were more likely to have myocardial ischemia as the reason for HTX (Table 1). All the other baseline anthropometric and clinical findings did not significantly differ between groups. As expected, more elevated plasma glucose and HbA1c levels in the T2DM than in non-T2DM patients at baseline were found (Table 1). After HTX, all patients were treated with ACE-I or ARB without differences among the groups (Table 1). Thus, the dosage of ACE-I (Lisinopril or Enalapril) or ARB (Losartan) was similar between the cohorts at baseline and at follow-up (Table 1). Anti-diabetic therapy of patients with pretransplant T2DM was reported in Table 1. None of the T2DM HTX had diabetic complications such as micro and macrovascular disease. Before HTX, T2DM patients evidenced optimal glucose and lipid control (Table 1). At 12-months follow-up, the T2DM patients were divided, as post-hoc analysis, into T2DM with good (HbA1c < 7%) and poor (HbA1c > 7%) glycemic control. Fifty-twoT2DM patients showed good glycemic control as evidenced by mean HbA1c < 7% (HbA1c 6.5 ± 0.3), assessed quarterly during the follow-up, and 38 T2DM patients showed poor glycemic control as evidenced by mean HbA1c ≥ 7% (HbA1c 8.2 ± 0.6) (Table 1). After HTX, the in-hospital echocardiographic evaluation showed a normal ejection fraction, slight alterations in the diastolic phase, and right ventricular function throughout the studied population without significant differences between T2DM and non-T2DM (Fig. 3A). After 12-months of follow-up, there was an impairment of both left and right ventricular function with a significant reduction of ejection fraction, Tricuspid Annular Plane Systolic Excursion (TAPSE), and E/e′ ratio in T2DM vs. non-T2DM recipients (p < 0.05) (Fig. 3A). Among diabetic patients, those with good glycemic control during the follow-up showed better cardiac function, both diastolic and systolic, than patients with poor glycemic control (Fig. 3B). Interestingly, correlation analysis evidences a relationship between diastolic and systolic changes and mean HbA1c levels (EF: R = − 0.423, P < 0.001; TAPSE: R = − 0.382, P < 0.001; E/e′ ratio: R = 0.341, P < 0.001). Moreover, there were no differences among patients treated with ACE-I and ARB (Additional file 1: Fig. S2). At 1-year follow-up, coronary CT angiography evaluations evidenced the absence of coronary lesions in the transplanted heart in T2DM and not-T2DM recipients (data not shown). Finally, myocardial perfusion by SPECT showed the absence of inducible ischemia in all patients (data not shown).

**Table 1:** Clinical characteristics of study population at 1 year of follow-up in ARNI users (n 106) vs. non-ARNI users’ patients (n 312)

	Basal	Follow-up	*P*	Basal	Follow-up	*P*	Basal	Follow-up	*P*
N	107	107	–	53	53	–	37	37	
Recipient data									
Mean age (years)	51.3 ± 5.9	–	–	50.5 ± 5.5	–	–	51.3 ± 5.5	–	–
Sex, male (%)	72 (67.3)	–	–	34 (64.1)	–	–	24 (64.9)	–	–
BMI (kg/m^2^)	25.5 ± 1.7	24.9 ± 1.4	0.010	27.8 ± 1.5*	26.2 ± 1.6*	0.001	27.9 ± 1.8*	27.4 ± 1.9*^§^	0.279
Aetiology of heart failure									
Myocardial ischemia, n (%)	50 (46.7)	–	–	27 (51.0)	–	–	19 (51.3)	–	–
Dilated cardiomyopathy, n (%)	44 (41.1)	–	–	20 (37.7)	–	–	14 (37.8)	–	–
Other, n (%)	13 (12.1)	–	–	6 (11.3)	–	–	4 (10.9)	–	–
Cardiovascular risk factors									
Hypertension, n (%)	33 (30.8)	–	–	25 (47.2)*	–	–	16 (43.2)*	–	–
Dyslipidemia, n (%)	21 (19.6)	–	–	16 (30.2)*	–	–	12 (32.4)*	–	–
Family history of CAD, n (%)	23 (21.5)	–	–	18 (33.9)*	–	–	13 (35.1)*	–	–
Smoking history, n (%)	13 (12.1)	–	–	8 (15.1)	–	–	5 (13.5)	–	–
Diabetes duration, years	–	–	–	14.6 ± 2.2	–	–	14.7 ± 3.1	–	–
Laboratory analyses									
Plasma glucose (mg/dl)	91.1 ± 6.4	91.7 ± 8.8	0.507	150.1 ± 12.1*	137.1 ± 7.2*	0.001	155.2 ± 26.2*	180.9 ± 25.4*^§^	0.001
HbA1c (%)	5.7 ± 0.9	5.8 ± 0.6	0.223	6.8 ± 1.2*	6.5 ± 0.4*	0.177	6.8 ± 1.1*	8.2 ± 0.6*^§^	0.001
Cholesterol (mg/dl)	159.8 ± 22.6	153.6 ± 17.4	0.013	173.9 ± 21.3*	164.5 ± 27.1*	0.039	177.1 ± 24.3*	183.7 ± 15.2*^§^	0.233
LDL-cholesterol (mg/dl)	94.5 ± 15.9	89.4 ± 14.6	0.016	95.8 ± 29.3	89.1 ± 13.8	0.141	97.2 ± 21.7	106.9 ± 14.9*^§^	0.671
HDL-cholesterol (mg/dl)	40.91 ± 2.5	40.3 ± 2.5	0.051	39.8 ± 3.5*	40.2 ± 2.9	0.536	38.8 ± 3.7*	38.3 ± 3.1*^§^	0.585
Triglycerides (mg/dl)	124.13 ± 28.1	119.5 ± 27.4	0.019	191.7 ± 22.4*	175.2 ± 21.4*	0.001	193.9 ± 22.5*	197.8 ± 15.2*	0.278
Creatinine (mg/dl)	1.1 ± 0.11	1.0 ± 0.44	0.421	1.1 ± 0.41	1.1 ± 0.81	0.523	1.1 ± 0.21	1.1 ± 0.58	0.446
Heart failure therapy									
ACEIs, n (%)	70 (65.4%)	71 (66.3)	0.236	33 (62.3%)	36 (67.9)	0.298	24 (64.9%)	24 (64.9%)	0.362
Lisinopril 5 mg, n (%)	9 (12.8%)	7 (9.9%)		4 (12.2%)	4 (11.1%)		3 (12.5%)	3 (12.5%)	
10 mg, n (%)	14 (20%)	18 (25.3%)		7 (21.3%)	8 (22.2%)		5 (20.8%)	6 (25%)	
20 mg, n (%)	11 (15.7%)	10 (14.1%)		5 (15.2%)	5 (13.8%)		3 (12.5%)	2 (8.3%)	
Enalapril 5 mg, n (%)	10 (14.3)	8 (11.3%)		5 (15.2%)	5 (13.8%)		4 (16.7%)	4 (16.7%)	
10 mg, n (%)	16 (22.9%)	19 (26.8%)		7 (21.3%)	10 (27.8%)		6 (25%)	7 (29.2%)	
20 mg, n (%)	10 (14.3%)	9 (12.7%)		5 (15.2%)	4 (11.1%)		3 (12.5%)	2 (8.3%)	
ARBs, n (%)	30 (28.1%)	36 (33.7)	0.457	12 (22.6%)	17 (32.1)	0.456	11 (29.7%)	13 (35.1)	0.473
Losartan 50 mg, n (%)	6 (20%)	6 (16.7%)		2 (16.7%)	2 (11.8%)		2 (18.2%)	2 (15.4%)	
100 mg, n (%)	15 (50%)	20 (55.5%)		6 (50%)	10 (58.8%)		5 (45.5%)	8 (61.6%)	
150 mg, n (%)	9 (30%)	10 (27.8%)		4 (33.3%)	5 (29.4%)		4 (36.4%)	3 (23.1%)	
Diuretics, n (%)	107 (100)	100 (93.4)	0.169	53 (100)	49 (92.4)	0.578	37 (100)	33 (89.1)	0.125
Beta-blokers	104 (97.2)	102 (95.3)	0.521	53 (100)	52 (98.1)	0.465	35 (94.6)	36 (97.3)	0.661
Calcium antagonists, n (%)	33 (30.8)	30 (28.1)	0.298	15 (28.3)	14 (26.4)	0.741	10 (27.1)	9 (24.3)	0.332
Anti-diabetic therapy									
Insulin, n (%)	–	–	–	10 (18.9)	12 (22.6)	0.560	8 (21.6)	11 (29.7)	0.282
Metformin, n (%)	–	–		42 (79.2)	46 (86.8)	0.451	30 (81.1)	32 (86.5)	0.457
DPP-IV inhibitor, n (%)	–	–	–	12 (22.6)	13 (24.5)	0.332	10 (27.2)	12 (32.4)	0.368
GLP-1 agonist, n (%)	–	–	–	7 (13.2)	6 (11.3)	0.599	4 (10.8)	5 (13.5)	0,446
Sulfonylureas, n (%)	–	–	–	4 (7.5)	0	/	2 (5.4)	0	–
Glinides, n (%)	–	–	–	6 (11.3)	2 (3.8)		4 (10.8)	5 (13.5)	
Donor data									
Mean age (years)	33.1 ± 9.9	–	–	32.4 ± 10.7	–	–	31.9 ± 10.6	–	–
Male, n (%)	50 (46.7)	–	–	24 (45.2)	–	–	18 (48.6)	–	–
BMI, kg/m^2^	26.1 ± 1.1	–	–	25.9 ± 1.4	–	–	26.4 ± 1.0	–	–
Donor ischemic time (min)	100.3 ± 19.9	–	–	101.1 ± 20.4	–	–	100.1 ± 13.6	–	–

Data are means ± SD or n (%)

*BMI* body mass index, *DPP-IV* dipeptidyl peptidase IV, *GLP-1* glucagon-like peptide-1

*P < 0.01 vs. non-diabetic patients. ^§^P < 0.01 vs. diabetic patients with HbA1c mean < 7%

### Expression of ACE2 and GlycACE2 in cardiomyocytes

**Fig. 4:**
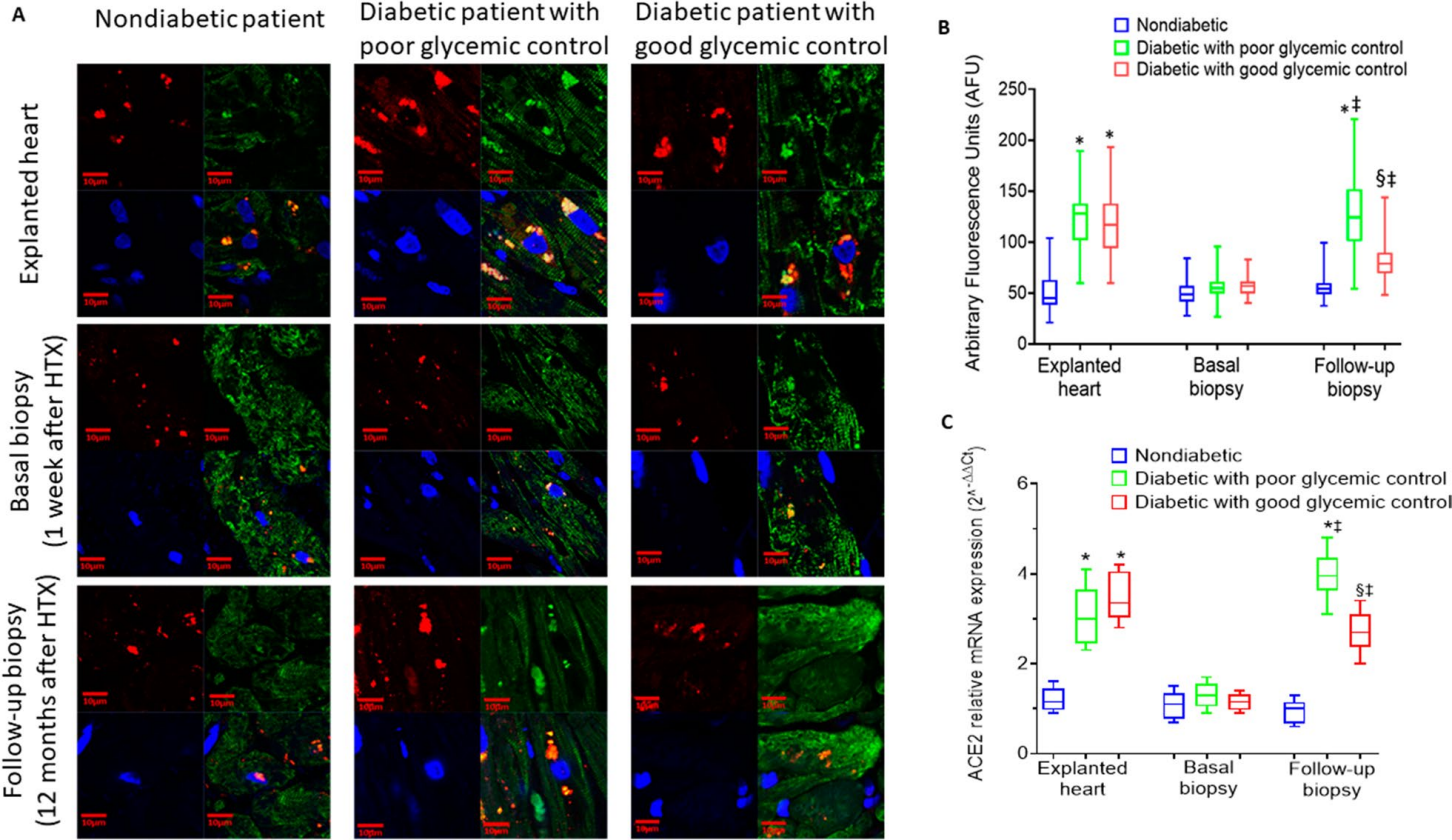
ACE2 protein levels in human cardiomyocytes. **A** Representative images and **B** analysis of ACE2 expression (red) and Cardiac Troponin T (green) in explanted heart tissue, basal and follow-up biopsies from nondiabetic and diabetic patients with poor or good glycemic control. Cell nuclei were stained blue with DAPI. Microscopy analyses were performed using an LSM 700 confocal microscope (Zeiss, Oberkochen, Germany) with a plan apochromat X63 (NA1.4) oil immersion objective. Fluorescence intensity analysis of myocardial ACE2 expression was estimated with ImageJ software and expressed as arbitrary fluorescence units (AFU). Data are presented as box and whisker plots showing medians (middle line) and the 2.5 and 97.5 percentiles using GraphPad Prism 9.1.2. software. **C** qRT-PCR analysis of ACE2 mRNA levels, expressed as 2^−ΔΔCt^ ± S.D., in human heart biopsies from nondiabetic patients (blue) and diabetic patients with poor (green) or good (red) glycemic control. *P < 0.05 vs Nondiabetic; ^‡^P < 0.05 vs Basal biopsy; § vs Diabetic with poor glycemic control at Follow-up biopsy

**Fig. 5:**
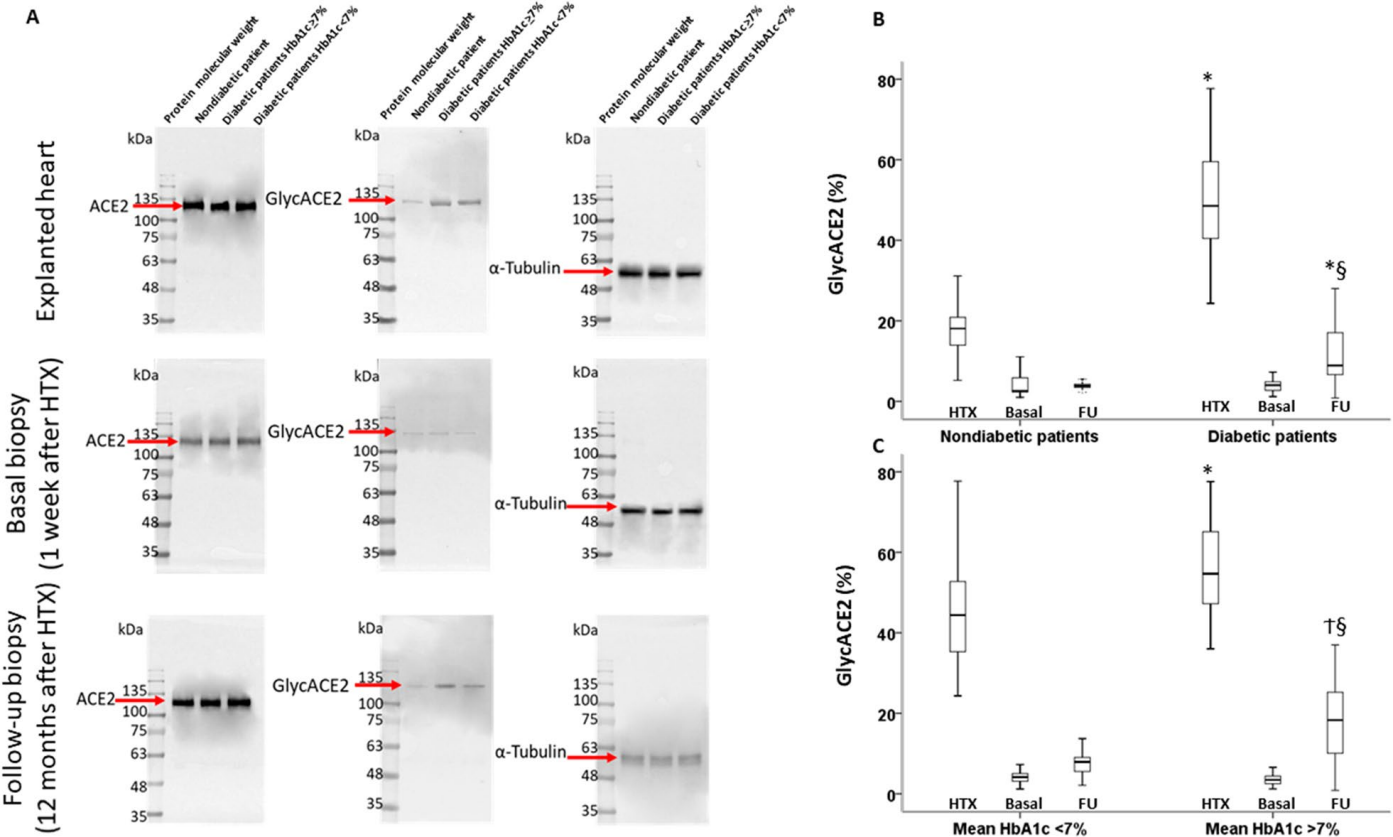
ACE2 and GlycACE2 expression in human cardiomyocytes. **A** Representative images of Western blotting analysis of ACE2 and GlycACE2 in explanted heart tissue, basal and follow-up biopsies from nondiabetic and diabetic patients with poor (HbA1c > 7%) or good (HbA1c < 7%) glycemic control. ACE2 and Glyc ACE2 levels were normalized using α-Tubulin. Glyc ACE2 levels in **B** nondiabetic and diabetic patients and **C** diabetic patients with HbA1c < 7% and diabetic patients with HbA1c > 7%. Analysis was performed by ImageJ 1.52n software; values were expressed as percentage and data were shown as box and whisker plots using GraphPad Prism 9.1.2. software. *P < 0.05 vs nondiabetic patients; ^†^P < 0.05 vs diabetic patients in good glycemic control (< 7%), ^§^P < 0.05 vs basal values

We first compared the myocardium of healthy transplanted hearts in recipients with and without T2DM by analyzing 394 EMBs for histological and molecular analyses until the 48th week after HTX. EMBs were divided into the following categories: basal (1 to 4 weeks) and final (44 to 48 weeks) (Fig. 1). Immunofluorescence and the Western Blot analyses (Figs. 4, 5) evidenced that in basal EMB, the expression of ACE2 in cardiomyocytes was similar among all recipient groups. At the final follow-up, immunofluorescence analysis evidenced that EMBs fromT2DMrecipients in poor glycemic control showed higher expression of ACE2 in cardiomyocytes than non-T2DM recipients and T2DM recipients in good glycemic control (Fig. 4). ACE2 gene levels also were increased in explanted heart of both diabetic patients with poor or glycemic control (respectively 2^−ΔΔCt^ = 3.1 ± 0.7 and 3.5 ± 0.6, both P < 0.05 vs nondiabetic) compared to nondiabetic patients (2^−ΔΔCt^ = 1.2 ± 0.3) (Fig. 4C). At follow-up, diabetic patients with poor glycemic control s showed an higher expression of ACE2 (2^−ΔΔCt^ = 3.9 ± 0.6, P < 0.05 vs nondiabetic at follow-up; P < 0.05 vs diabetic with poor glycemic control at basal biopsy), as well as diabetic with high glycemic control (2^−ΔΔCt^ = 2.7 ± 0.5, P < 0.05 vs nondiabetic at follow-up; P < 0.05 vs diabetic with high glycemic control at basal biopsy) (Fig. 4C). However, diabetic patients with high glycemic control showed a significant reduction of ACE2 gene levels (P < 0.05) at follow-up compared to diabetic patients with poor glycemic control (Fig. 4C). Evaluation of ACE2 and GlycACE2 expression levels showed a remarkably higher percentage of GlycACE2 expression in T2DM EMBs, whereas low levels of GlycACE2 were observed in the EMBs from non-T2DM patients (Fig. 5). Interestingly, among diabetic patients, those with good glycemic control during the follow-up showed a lower percentage of both ACE2 and GlycACE2 than patients with poor glycemic control (Fig. 5). Accordingly, correlation analysis evidences a relationship betweenGlycACE2 and BMI at follow-up and mean HbA1c levels during follow-up (R = 0.706, P < 0.001).In the multiple regression model, changes in GlycACE2 levels were independent of age, gender, hypertension, and dyslipidemia (Additional file 1: Table S1). Finally, there were no differences among patients treated with ACE-I and ARB (Additional file 1: Fig. S3).

### Ang 1–9, Ang 1–7, MasR and NAFT in the cardiac biopsies

**Fig. 6:**
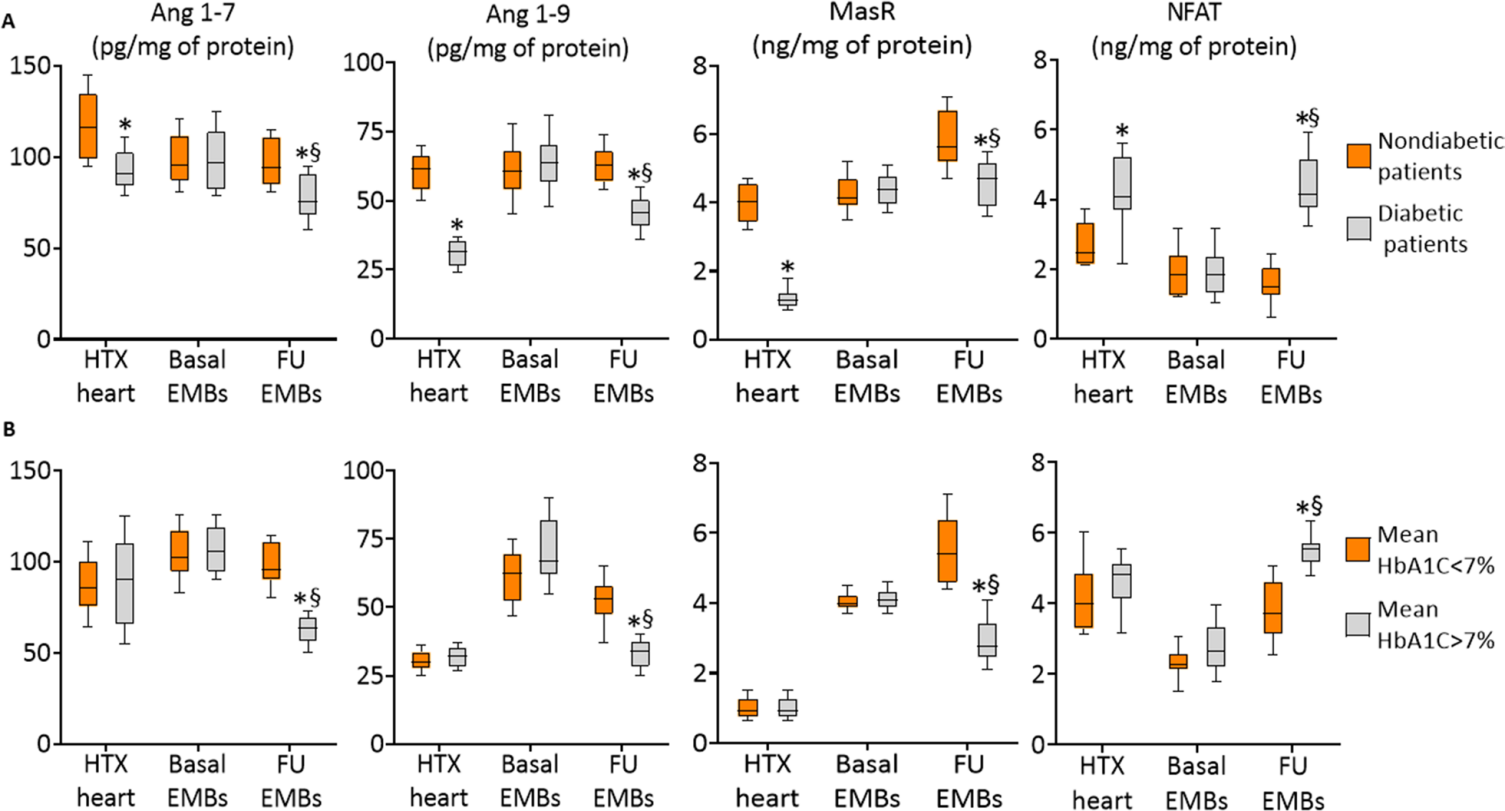
**A** Evaluation of Angiotensin-1–9 (Ang-1–9), Ang 1–7, MAS1, and NFAT activation molecule 1 (NFAM1) content in explanted hearts (HTX) at week 1 (Basal) and week 48 (follow-up) from HTX, in nondiabetic and diabetic patients. *P < 0.05 vs nondiabetics, ^§^P < 0.05 vs basal values. **B** Levels of Ang-1–9, Ang 1–7, MAS1, and NFAM1 assessed in explanted hearts (HTX) at week 1 (Basal) and week 48 (Follow-up) from HTX, in the diabetic patients with good glycemic control (HbA1c < 7%) and diabetic patients with poor glycemic control (HbA1c ≥ 7%) *P < 0.05 vs diabetic patients in good glycemic control (< 7%), ^§^P < 0.05 vs basal values

To evaluate the activity of GlycACE2, we analyzed Ang 1–9, Ang 1–7, MasR, and NAFT in T2DM and non-T2DMventricular specimens (Fig. 6). Ang 1–9, Ang 1–7, MasR, and NAFT expressions in heart EMBs of the basal period showed similar levels in both T2DM and non-T2DM recipients. However, at follow-up, Ang 1–9, Ang 1–7, and MasR expressions in ventricular specimens from T2DM patients were lower, whereas NAFT expressions were higher than non-T2DM ventricular specimens (Fig. 6). Interestingly, among T2DM patients, those with good glycemic control during the follow-up showed a higher amount of Ang 1–9, Ang 1–7, and MasR, and a lower amount of NAFT than patients with poor glycemic control (Fig. 6). Remarkably, Ang 1–9, Ang 1–7, and MasR levels in ventricular specimens were inverse,whereas NAFT levels were directly related to GlycACE2 levels(Ang 1–7: R = − 0.844, P < 0.001; Ang 1–9: R = − 0.762, P < 0.001; MasR: R = − 0.613, P < 0.001; NAFT: R = 0.702, P < 0.001). Interestingly, there were no differences among patients treated with ACE-I and ARB (Additional file 1: Fig. S4A, B). Finally, the urinary levels of Ang 1–9 and Ang 1–7 were not different in T2DM patients than in non-T2DM patients at baseline (T2DM 159.6 ± 52 pg/ml, non-T2DM:161.7 ± 71 pg/ml, P < 0.121). However, the urinary levels of both Ang 1–9 and Ang 1–7 were lower in T2DM patients with poor glycemic control at follow-up than both non-T2DM or T2DM patients with good glycemic control at follow up (T2DM with poor glycemic control 61.7 ± 45 pg/ml, T2DM with good glycemic control 166.1 ± 475 pg/ml, non-T2DM:159.1 ± 39 pg/ml, P < 0.01 for both).

### Fibrosis in cardiac biopsies

**Fig. 7:**
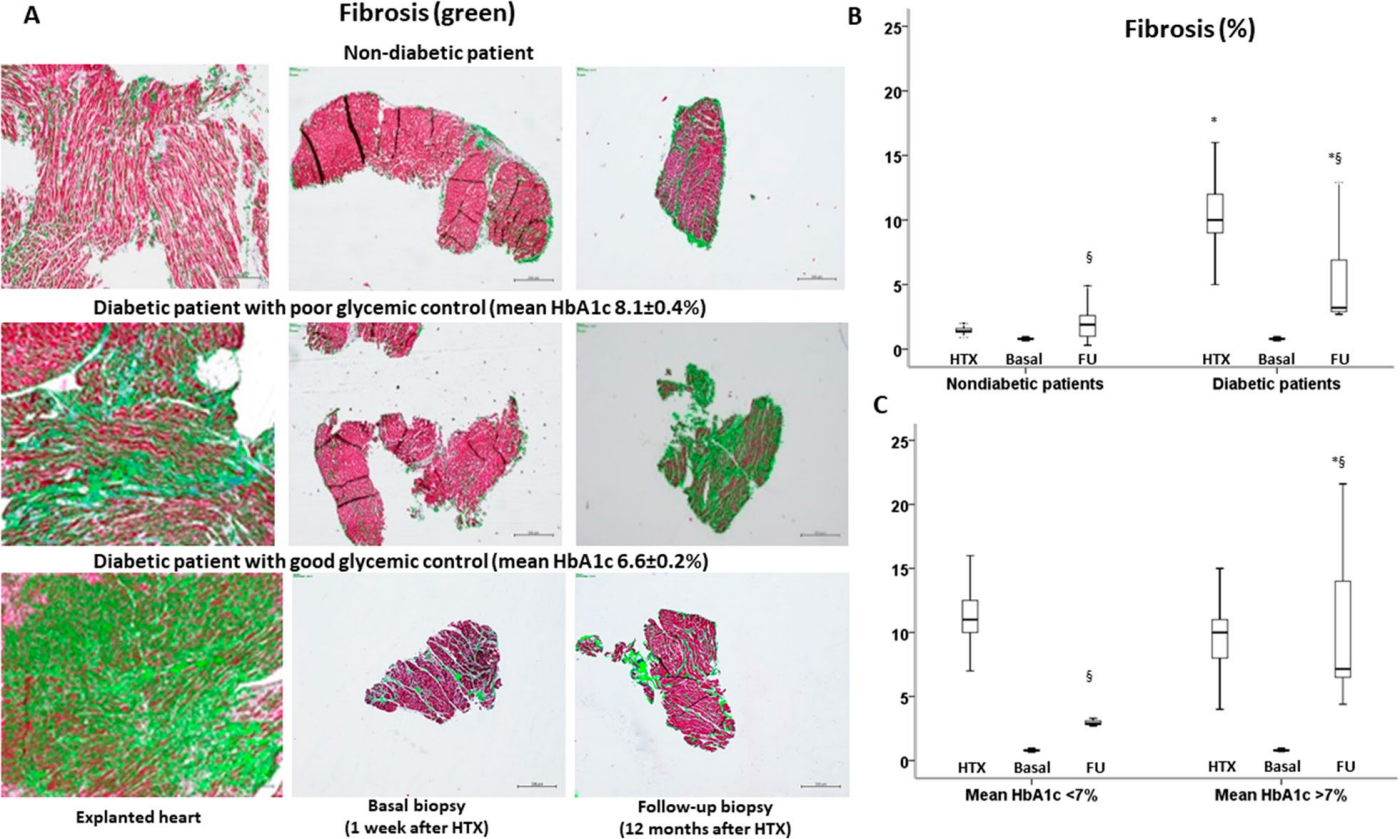
**A** Fibrosis (green) in heart specimens from nondiabetic patient, diabetic patient with poor glycemic control (mean HbA1c 8.1 ± 0.4%), and diabetic patient with good glycemic control (mean HbA1c 6.6 ± 0.2%). **B** Percent values of fibrosis (ZEN 2.5 pro software) in explanted hearts (HTX) at week 1 (Basal) and week 48 (Follow-up) from HTX in nondiabetic and diabetic patients. P < 0.05 vs nondiabetics, ^§^P < 0.05 vs basal values. **C** Percent values of fibrosis (ZEN 2.5 pro software) in explanted hearts (HTX) at week 1 (Basal) and week 48 (Follow-up) from HTX, in the diabetic patients with good glycemic control (HbA1c < 7%) and diabetic patients with poor glycemic control (HbA1c ≥ 7%) *P < 0.05 vs diabetic patients in good glycemic control (< 7%), ^§^P < 0.05 vs basal values

Because the main effect of Ang 1–9 and Ang 1–7 is the antifibrotic heart remodeling through MasR increased activity and reduced NAFT expression, ventricular specimens of T2DM and non-T2DM patients for the occurrence of fibrosis were also analyzed (Fig. 7). Fibrosis expressions in heart EMBs of the basal period showed similar levels in both subgroups of T2DM and non-T2DM recipients. However, at follow-up, immunohistochemistry evidenced that fibrosis levels in ventricular specimens from T2DM patients were higher than non-T2DM ventricular specimens in both ACE-I and ARB-treated patients (Fig. 7). Interestingly, among diabetic patients, those with good glycemic control during the follow-up showed a lower amount of fibrosis than patients with poor glycemic control in both ACE-I and ARB-treated patients (Fig. 7). Remarkably, Ang 1–9, Ang 1–7, and MasR in ventricular specimens were inversely related to fibrosis levels (Ang 1–7: R = − 0.705, P < 0.001; Ang 1–9: R = − 0.666, P < 0.001; MasR: R = 0.599, P < 0.001). On the other hand, mean HbA1c, GlycACE2 and NAFT expressions were directly related to fibrosis (mean HbA1c: R = 0.648, P < 0.001; NAFT: R = 0.653, P < 0.001**)**. Finally, there were no differences among patients treated with ACE-I and ARB (Additional file 1: Fig. S5).

### Findings in resident explanted hearts with end-stage of HF

Although the analysis of explanted resident hearts provided no new information on the progression of DCM, higher expressions of ACE2 and GlycACE2 were observed in explanted hearts from T2DM patients regardless of the cause of HF (Figs. 4 and 5) to get insight into the pathophysiology of end-stage HFrEF along with diabetes, as previously described [14]. In addition, in this study, we evidenced that Ang 1–9, Ang 1–7, and MasR were reduced. At the same time, NAFT expressions increased in cardiomyocytes of explanted hearts of T2DM compared to non-T2DMpatients (P < 0.05), thus indicating more negligible cardioprotective effects by RAS-inhibition in diabetic end-stage HF (Fig. 6). Finally, fibrosis levels were higher in T2DM than in non-T2DM explanted hearts regardless of the cause of HF (Fig. 7).

## Discussion

Our data demonstrate that poor glycaemic results in increased myocardial levels of the GlycACE2, with a concomitant reduction of the cardiac protection of the RAS-inhibition in humans hearts, thus attenuating the cardioprotective effects of the RAS inhibition in human beating hearts. More specifically, we documented that: (1) higher level of GlycACE2 expression in cardiomyocytes of TD2M correlated with glycemic control expressed as HbA1c levels; (2) non-enzymatic glycosylation can affect GlycACE2 levels with concomitant impairment of ACE2 activity in cardiac biopsies from T2DM patients, as evidenced by the reduced levels of Ang 1–9, Ang 1–7, and MasR; (3) the specific pathogenic contribution of GlycACE2 correlated with myocardial fibrosis and development of impaired heart function; and (4) good glycemic control, as a mean HbA1c of < 7% during 12 months after HTX, improved the anti-remodeling effects of the RAS inhibition by reducing myocardial levels of GlycACE2 as well as increasing levels of Ang 1–9, Ang 1–7, and MasR. Moreover, regardless of the pathogenic causes of HF, explanted hearts from T2DM patients had high levels of GlycACE2 and fibrosis and reduced levels of Ang 1–9, Ang 1–7, and MasR (Central illustration).

Experimental studies evidenced that Ang 1–7 treatment ameliorated left ventricular remodeling and dysfunction in diabetic rats by attenuating myocardial fibrosis, myocardial hypertrophy, and myocyte apoptosis via the MasR/NAFT pathways [20, 21]. Furthermore, Ang 1–7 combined with perindopril provided additional cardioprotection towards single therapy, suggesting a reduced anti-remodeling effect of RAS-inhibition therapy in hyperglycemic rats. Our unique ongoing real-life study investigated the early detrimental effects of poor glycemic on the molecular mechanisms involved in the cardioprotective effects of RAS-inhibition, such as in healthy heart transplanted in T2DM recipients. According to previous evidence [14] the diabetic milieu favored non-enzymatic glycosylation of myocardial ACE2 proteins highly dependent on HbA1c levels. Protein glycation and the formation of advanced glycation end products play an important role in the pathogenesis of diabetic complications and other chronic diseases encompassing rheumatoid arthritis or osteoporosis, as well as in aging [22, 23] and also contribute to the epigenetic-sensitive related diabetic complications [24]. Within this framework, the novelty of this study is represented by the evidence that high levels of myocardial GlycACE2 in T2DM patients with poor glycemic control may impair the effects of RAS inhibition and the response to ACE-I and ARB therapy. Within the RAS system, the ACE2/Ang 1–7/Ang 1–9/MasR axis counterposes the ACE1/Ang II/AT1 receptor axis. With a homologous catalytic domain as for ACE1, ACE2 competes with ACE1 to convert Ang II to Ang 1–7 and Ang 1–9, which provides anti-vasoconstrictive, anti-inflammatory, anti-hypertrophic, and antifibrotic effects on various tissue including the cardiomyocytes by inhibiting NFAT [25, 26]. Thus, inhibiting the RAS and activating the counterbalancing ACE2/Ang 1–7/Ang 1–9/MasR axis might have complementary action in cardiovascular diseases, including heart failure progression [27]. However, our study allows a cautious optimism regarding adequate cardiac protection of RAS-inhibition in patients with diabetes, suggesting that achievement of tight glycemic control normalizes the anti-remodeling effects of ACE-I and ARB therapy. Accordingly, 1 year of good glycemic control, as evidenced by the quarterly evaluated mean value of HbA1c < 7%, was associated with reduced GlycACE2 and consequently with improved ACE2/Ang 1–7 1–9/Mas axis as well as with reduced NFAT expression and fibrosis. Urinary levels of both Ang 1–9 and Ang 1–7 were not different in T2DM patients than in non-T2DMpatients at baseline when all diabetic patients have good glycemic control. Remarkably, the urinary levels of both Ang 1–9 and Ang 1–7 decreased only in T2DM patients with poor glycemic control at follow-up, indicating a causal relationship between the activity of ACE2 and high glucose levels, likely through the occurrence of non-enzymatic glycosylation of ACE2 protein. Indeed, our data in resident failing hearts who were treated with RAS-inhibition, evidenced higher myocardial GlycACE2, NFAT and cardiac fibrosis along with reduced levels of Ang 1–7, Ang 1–9, and MasR supporting an impaired cardioprotective effect of RAS-inhibition in diabetic patients with severe HF compared to nondiabetic patients. Moreover, our study evidenced that RAS inefficacy is a pathogenic event unrelated to the other pathogenic events of end-stage HF (ischemic, idiopathic, infective, and rheumatic) [28]. Furthermore, our data suggest that RAS inefficiency begins early in transplanted healthy hearts of T2DM but not in non-T2DMrecipients, as proved by increase of GlycACE2, NFAT, and fibrosis and decrease of Ang 1–7, Ang 1–9, and MasR already after 12-months from HTX but not during the first EMBs after HTX. Therefore, the diabetic milieu can promptly alter the ACE2 activity. In fact, Ang 1–7, Ang 1–9, and MasR myocardial contents were related to systemic glycemic control, as evidenced by regression analysis. Moreover, we showed that Ang 1–7, Ang 1–9, and MasR decreases in T2DM patients were associated with cardiac fibrosis, independently of BMI, heart rate, and blood pressure. Ang 1–7, Ang 1–9, and MasR were related to early diastolic, and systolic dysfunction observed in T2DMrecipients after 12-months from HTX. Furthermore, these alterations were independent of CHD (as showed by negative coronary CT angiography and ECG stress test).

### Study limitations

First, our real-life study was based at only a single institution and had a small number of subjects, so we need to extend our observations to a larger cohort of patients. Second, immunosuppressive therapy per se could affect the molecular mechanisms of RAS inhibition. However, regression analysis showed that progressive decreases of ANG 1–7, ANG 1–9, and MasR in T2DM recipients were independent of the immunosuppressive state, including covariables such as polyclonal anti-lymphocyte antibodies, cyclosporine, tacrolimus, mycophenolate mofetil, everolimus, and prednisone. Moreover, the findings that higher levels of GlycACE2 and reduced ACE2/Ang 1–7, 1–9/MasR axis with a parallel increase of myocardial fibrosis were observed only in diabetic patients, as well as the improvement of anti-remodeling effects of RAS inhibition in diabetic patients with HbA1c levels < 7% suggest a pivotal role of glycemic control in the efficacy of both ACE-I and ARB therapy. Third, our molecular data come from cross-sectional analyses.

Finally, in the current study we did not measure the plasma renin activity (PRA) levels after the treatment with ARB or ACE-I. Despite this, the PRA levels and consequently the PRA-guided therapy have been proposed to evaluate the effects of the ACE-I or ARB on the treated patients [27].

## Conclusions

Poor glycemic control may favor glycation of ACE2 and reduce the cardiac protection of RAS-inhibition. Furthermore, our study suggests that the achievement of tight glycemic control normalizes there modeling effects of ACE-I and ARB therapy. Finally, our data assume an important role in understanding the effects of RAS-inhibition on the molecular mechanisms of the diabetic cardiomyocyte, in light of recent evidence suggesting that the RAS should be regarded as both a circulating and cellular organized hierarchical angiotensin network linked by characteristic enzymatic reactions [27, 28].

## Perspectives

Competency in patient care and procedural outcomes: good glycemic control, as evidenced by HbA1c levels < 7%, improves anti-remodeling effects of RAS-inhibition therapy in diabetic patients.

Translational outlook: longer-term follow-up and direct comparisons with other therapy as sacubitril/valsartan will help define the role of anti-remodeling therapy to prevent diabetic cardiomyopathy.

## Supplementary Information

**Additional file 1: Figure S1.** Western blot analysis of GlycACE2. Representative immunoblotting of recombinant hACE2 protein (ab151852, Abcam) after in vitro long-term exposure to glucose 120 mM was separated on SDS-PAGE by using 7% gels in reducing and non-reducing conditions and then transferred on nitrocellulose membrane. Membrane was incubated with specific primary antibody against GlycACE2 (1:1000) (#4355, Cell Signaling Technology). Molecular weight indicators are displayed at the center. **Figure S2.** Ejection fraction, TAPSE, and the E/e′ at week 1 (Basal) and week 48 (follow-up) in nondiabetic and diabetic patients treated with ACE-inhibitors (ACE-I) and angiotensin receptor blocker (ARB). Data are mean ± SD. *P < 0.05 vs non-diabetics, ^§^P < 0.05 vs basal values. **Figure S3.** GlycACE2 at week 1 (Basal) and week 48 (Follow-up) in the diabetic patients with good glycemic control (HbA1c < 7%) and diabetic patients with poor glycemic control (HbA1c ≥ 7%) treated with ACE-inhibitors (ACE-I) and angiotensin receptor blocker (ARB). Data are mean ± SD. *P < 0.05 vs non-diabetics, §P < 0.05 vs basal values. **Figure S4.** **A** Angiotensin-1–9 (Ang-1–9), Ang 1–7, Mas receptor (MasR), Nuclear factor of activated T-cells (NFAT), in explanted hearts (HTX) at week 1 (Basal) and week 48 (Follow-up) from HTX, in nondiabetic and diabetic patients, treated with ACE-inhibitors (ACE-I) and angiotensin receptor blocker (ARB). **B** Ang-1–9, Ang 1–7, MasR, and NFAT, in explanted hearts (HTX) at week 1 (Basal) and week 48 (Follow-up) from HTX,in the diabetic patients with good glycemic control (HbA1c < 7%) and diabetic patients with poor glycemic control (HbA1c ≥ 7%) treated with ACE-inhibitors (ACE-I) and angiotensin receptor blocker (ARB). Data are mean ± SD. *P < 0.05 vs non-diabetics, ^§^P < 0.05 vs basal values. **Figure S5.** Fibrosis percentage in explanted hearts (HTX), at week 1 (Basal) and week 48 (follow-up) from HTX, in the diabetic patients with good glycemic control (HbA1c < 7%) and diabetic patients with poor glycemic control (HbA1c ≥ 7%) treated with ACE-inhibitors (ACE-I) and angiotensin receptor blocker (ARB). Data are mean ± SD. *P < 0.05 vs non-diabetics, ^§^P < 0.05 vs basal values. **Table S1.** Multivariate linear regression analysis with GlycACE2 as dependent variable.

## Data Availability

Data and materials are full available on demand.

## Abbreviations

ADA: American Diabetes Association
ALCOA: Attributable, Legible, Contemporaneous, Original and Accurate
ACE-I: Angiotensin-converting enzyme inhibitor
ACE2: Angiotensin-converting enzyme 2
Ang 1–7: Angiotensin-1–7
Ang 1–9: Angiotensin-1–9
ARB: Angiotensin receptor blocker
DSA: Donor-specific antibodies
EF: Ejection fraction
EMB: Endomyocardial biopsy
GlycACE2: Glycosylated ACE2
HbA1c: Glycated hemoglobin
HTX: Heart transplanted
ISHLT: International Society for Heart Lung Transplantation
IVS: Interventricular septum
MasR: Mas receptor
NFAT: Nuclear factor of activated T-cells
RAS: Renin-angiotensin system
TAPSE: Tricuspid Annular Plane Systolic Excursion
T2DM: Type 2 diabetes mellitus
