# Supplementary material for: Glycated ACE2 reduces anti-remodeling effects of renin-angiotensin system inhibition in human diabetic hearts
Source: Cardiovasc Diabetol. 2022 Aug 5;21:146. doi: 10.1186/s12933-022-01573-x (PMC9356400; doi:10.1186/s12933-022-01573-x)
Supplement: Supplementary file 1 — Additional file 1: Figure S1. Western blot analysis of GlycACE2. Representative immunoblotting of recombinant hACE2 protein (ab151852, Abcam) after in vitro long-term exposure to glucose 120 mM was separated on SDS-PAGE by using 7% gels in reducing and non-reducing conditions and then transferred on nitrocellulose membrane. Membrane was incubated with specific primary antibody against GlycACE2 (1:1000) (#4355, Cell Signaling Technology). Molecular weight indicators are displayed at the center. Figure S2. Ejection fraction, TAPSE, and the E/e′ at week 1 (Basal) and week 48 (follow-up) in nondiabetic and diabetic patients treated with ACE-inhibitors (ACE-I) and angiotensin receptor blocker (ARB). Data are mean ± SD. *P < 0.05 vs non-diabetics, §P < 0.05 vs basal values. Figure S3. GlycACE2 at week 1 (Basal) and week 48 (Follow-up) in the diabetic patients with good glycemic control (HbA1c < 7%) and diabetic patients with poor glycemic control (HbA1c ≥ 7%) treated with ACE-inhibitors (ACE-I) and angiotensin receptor blocker (ARB). Data are mean ± SD. *P < 0.05 vs non-diabetics, §P < 0.05 vs basal values. Figure S4. A Angiotensin-1–9 (Ang-1–9), Ang 1–7, Mas receptor (MasR), Nuclear factor of activated T-cells (NFAT), in explanted hearts (HTX) at week 1 (Basal) and week 48 (Follow-up) from HTX, in nondiabetic and diabetic patients, treated with ACE-inhibitors (ACE-I) and angiotensin receptor blocker (ARB). B Ang-1–9, Ang 1–7, MasR, and NFAT, in explanted hearts (HTX) at week 1 (Basal) and week 48 (Follow-up) from HTX,in the diabetic patients with good glycemic control (HbA1c < 7%) and diabetic patients with poor glycemic control (HbA1c ≥ 7%) treated with ACE-inhibitors (ACE-I) and angiotensin receptor blocker (ARB). Data are mean ± SD. *P < 0.05 vs non-diabetics, §P < 0.05 vs basal values. Figure S5. Fibrosis percentage in explanted hearts (HTX), at week 1 (Basal) and week 48 (follow-up) from HTX, in the diabetic patients with good glycemic control (HbA1c < 7%) and diabe [file 12933_2022_1573_MOESM1_ESM.docx]

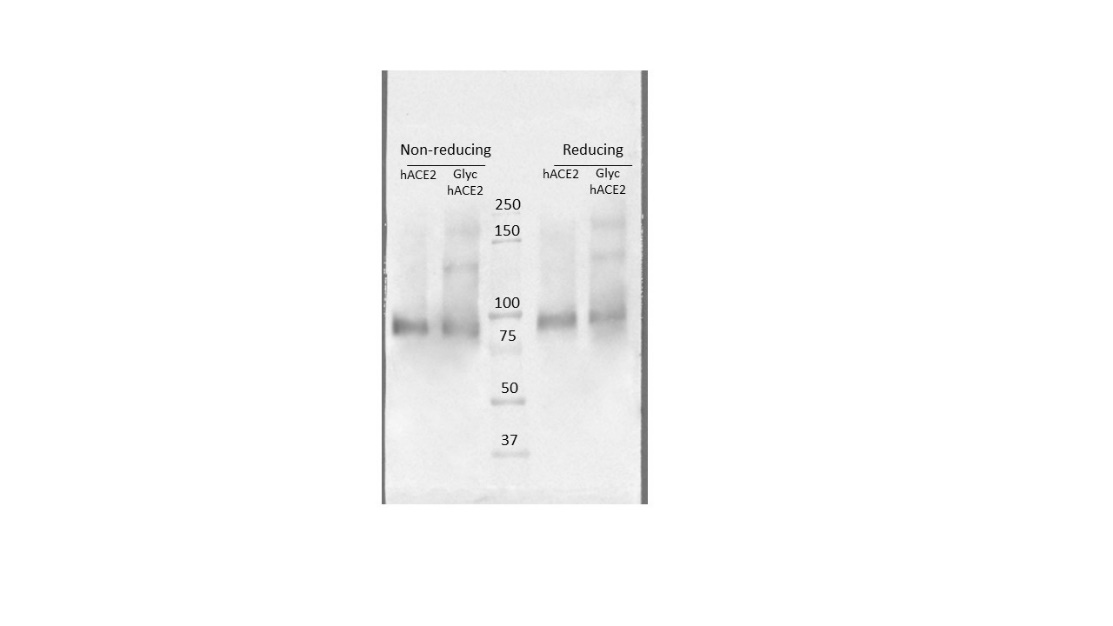


**Figure S1.** Western blot analysis of GlycACE2. Representative immunoblotting of recombinant hACE2 protein (ab151852, Abcam) after in vitro long-term exposure to glucose 120 mM was separated on SDS-PAGE by using 7% gels in reducing and non-reducing conditions and then transferred on nitrocellulose membrane. Membrane was incubated with specific primary antibody against GlycACE2 (1:1000) (#4355, Cell Signaling Technology). Molecular weight indicators are displayed at the center.

**
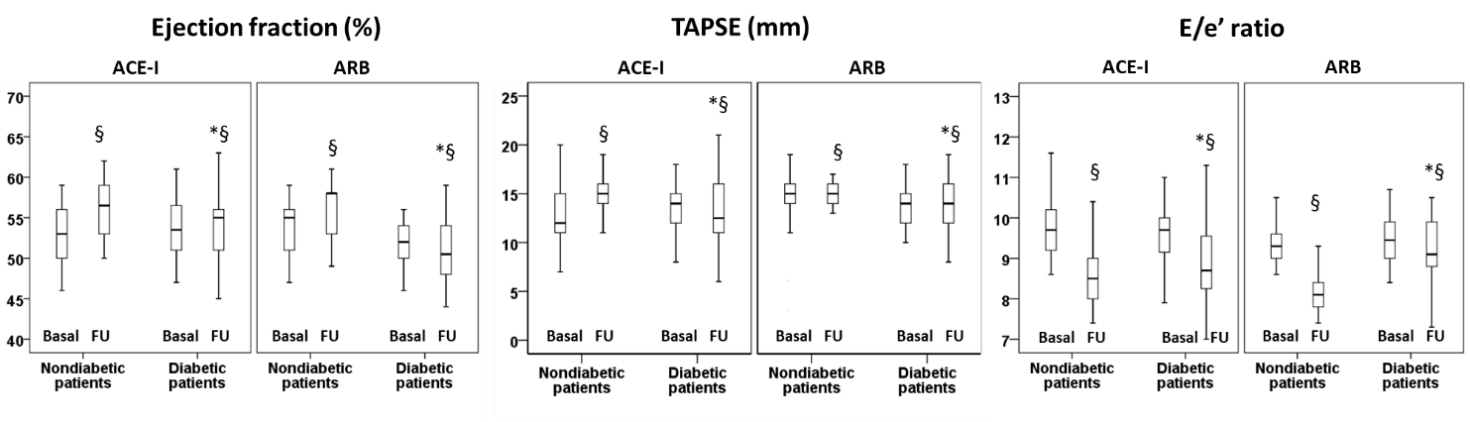
**

**Figure S2.** Ejection fraction, TAPSE, and the E/e′ at week 1 (Basal) and week 48 (Follow-up) in nondiabetic and diabetic patients treated with ACE-inhibitors (ACE-I) and angiotensin receptor blocker (ARB). Data are mean ±SD. *P<0.05 vs non-diabetics, §P<0.05 vs basal values.


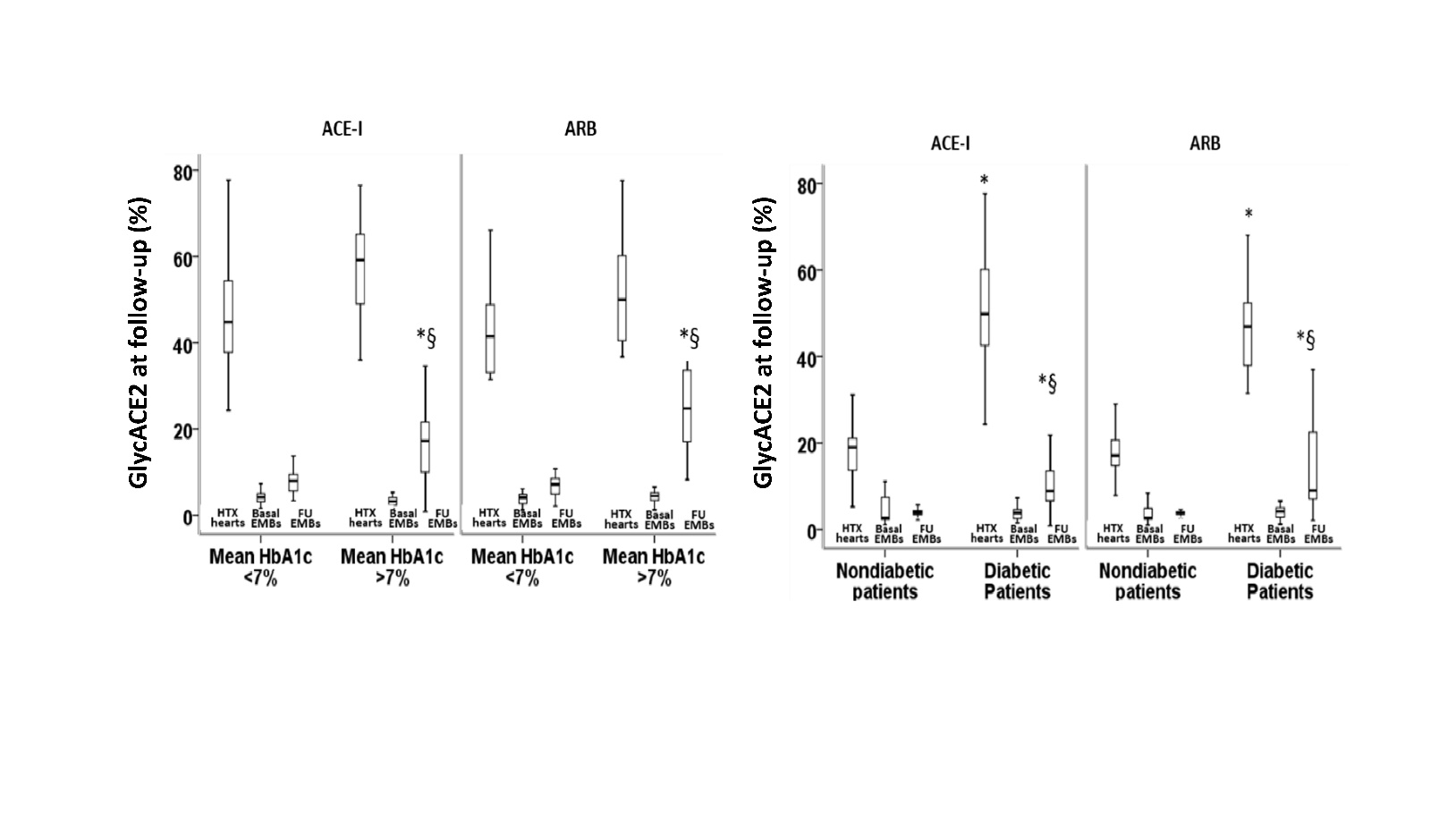


**Figure S3.** GlycACE2 at week 1 (Basal) and week 48 (Follow-up) in the diabetic patients with good glycemic control (HbA1c <7%) and diabetic patients with poor glycemic control (HbA1c >7%) treated with ACE-inhibitors (ACE-I) and angiotensin receptor blocker (ARB). Data are mean ±SD. *P<0.05 vs non-diabetics, §P<0.05 vs basal values.

**
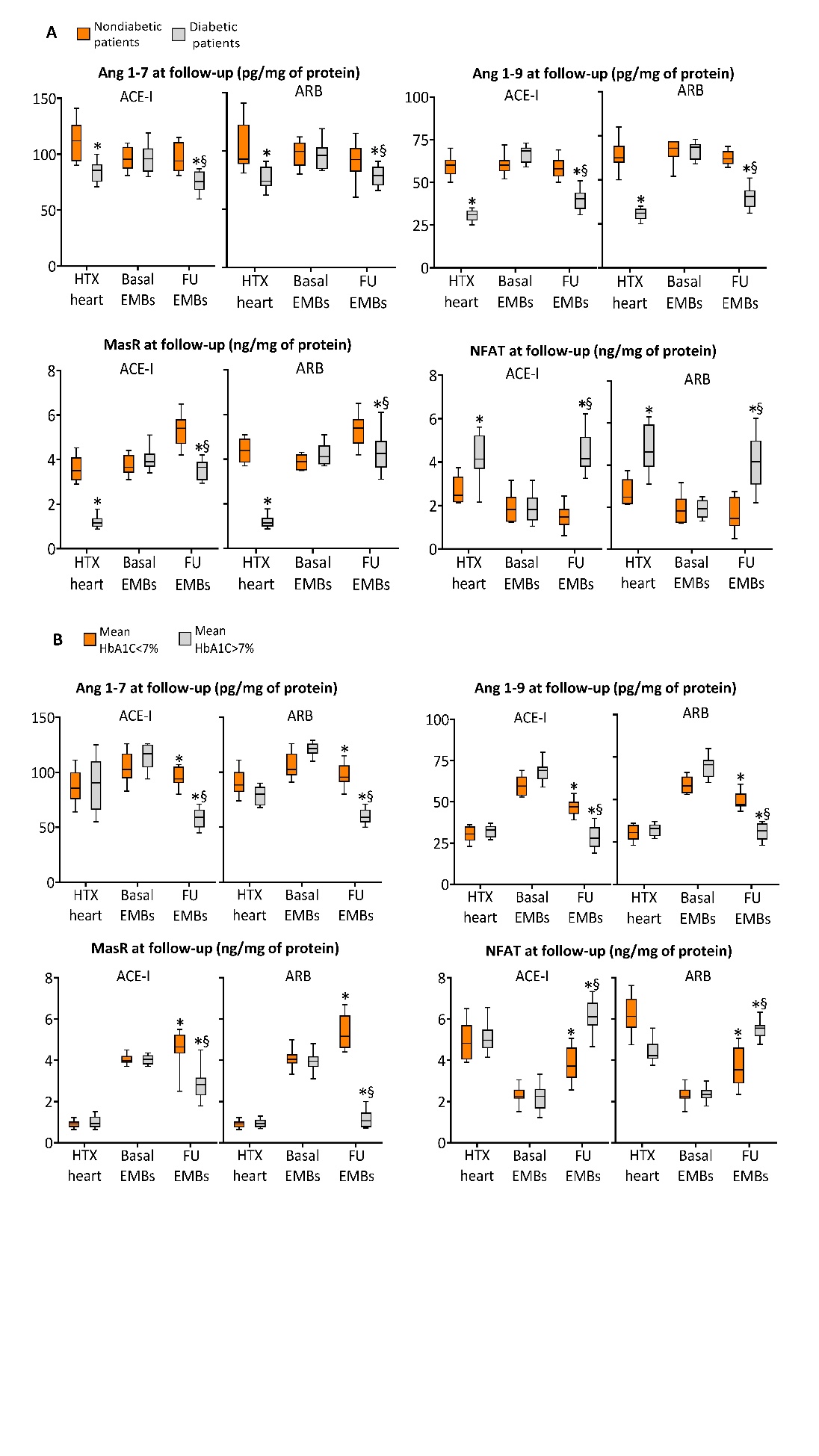
**

**Figure S4. Panel A,** Angiotensin-1-9 (Ang-1-9), Ang 1-7, Mas receptor (MasR), Nuclear factor of activated T-cells (NFAT), in explanted hearts (HTX) at week 1 (Basal) and week 48 (Follow-up) from HTX, in nondiabetic and diabetic patients, treated with ACE-inhibitors (ACE-I) and angiotensin receptor blocker (ARB). **Panel B,** Ang-1-9, Ang 1-7, MasR, and NFAT, in explanted hearts (HTX) at week 1 (Basal) and week 48 (Follow-up) from HTX,in the diabetic patients with good glycemic control (HbA1c <7%) and diabetic patients with poor glycemic control (HbA1c >7%) treated with ACE-inhibitors (ACE-I) and angiotensin receptor blocker (ARB). Data are mean ±SD. *P<0.05 vs non-diabetics, §P<0.05 vs basal values.


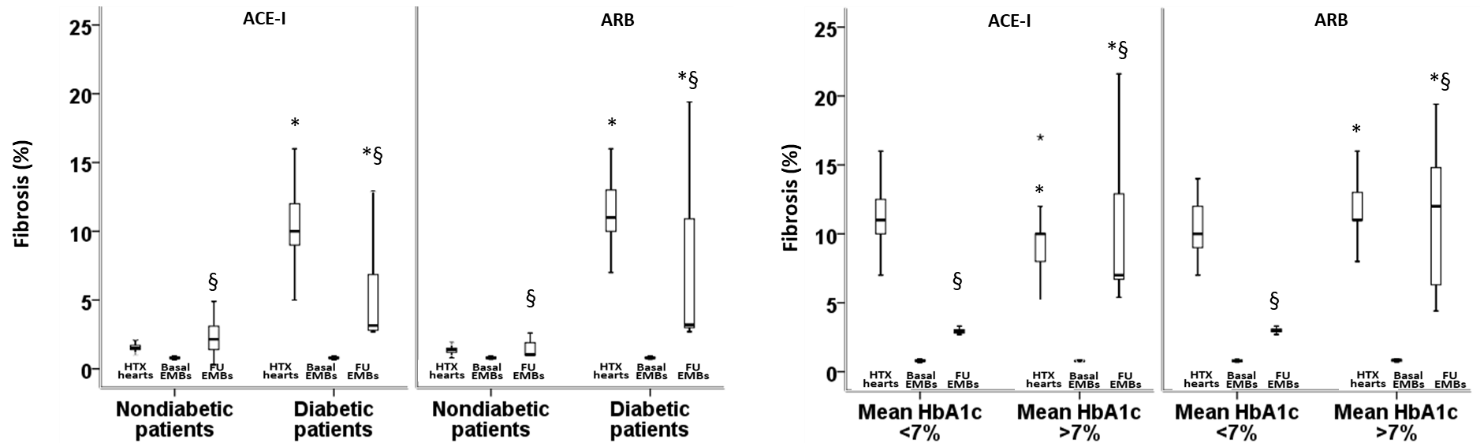


**Figure S5.** Fibrosis percentage in explanted hearts (HTX), at week 1 (Basal) and week 48 (Follow-up) from HTX, in the diabetic patients with good glycemic control (HbA1c <7%) and diabetic patients with poor glycemic control (HbA1c >7%) treated with ACE-inhibitors (ACE-I) and angiotensin receptor blocker (ARB). Data are mean ±SD. *P<0.05 vs non-diabetics, §P<0.05 vs basal values.

**Table S1.** Multivariate linear regression analysis with GlycACE2 as dependent variable

|  |  |  | **Confidence** | **Interval 95%** |
| --- | --- | --- | --- | --- |
| **Variables** | **Beta** | **P** | **Lower Limit** | **Upper Limit** |
| **Age** | 0.016 | 0.757 | -0.106 | 0.145 |
| **Male** | 0.029 | 0.589 | -1.107 | 1.942 |
| **Basal BMI** | -0.144 | 0.021 | -0.925 | -0.076 |
| **Hypertension** | 0.027 | 0.605 | -1.192 | 2.040 |
| **Dyslipidemia** | 0.014 | 0.809 | -2.170 | 1.695 |
| **Mean HbA1c** | 0.785 | 0.001 | 3.360 | 4.549 |
